# Age and estimated glomerular filtration rate in Chinese older adults: a cohort study from 2014 to 2020

**DOI:** 10.3389/fpubh.2024.1392903

**Published:** 2024-06-25

**Authors:** Ying Jiang, Qin Cao, Weiqi Hong, Tianwei Xu, Molian Tang, Yun Li, Renying Xu

**Affiliations:** ^1^Department of Clinical Nutrition, Ren Ji Hospital, Shanghai Jiao Tong University School of Medicine, Shanghai, China; ^2^Health Management Center, Ren Ji Hospital, Shanghai Jiao Tong University School of Medicine, Shanghai, China; ^3^Shanghai Pudong New Area Caolu Community Health Center, Shanghai, China; ^4^Stress Research Institute, Department of Psychology, Stockholm University, Stockholm, Sweden; ^5^School of Public Health, North China University of Science and Technology, Tangshan, China; ^6^Department of Nutrition, College of Health Science and Technology, Shanghai Jiao Tong University School of Medicine, Shanghai, China

**Keywords:** aged, glomerular filtration rate, obesity, blood pressure, dyslipidemias

## Abstract

**Objectives:**

This study aimed to fill the data gap of the course of renal function decline in old age and explore changes in renal function across different health states with increasing age.

**Methods:**

This observational, retrospective, single-center cohort study included 5,112 Chinese older adults (3,321 men and 1,791 women, range 60–104 years). The individual rate of estimated glomerular filtration rate (eGFR) decline was analyzed using linear mixed-effects model to account for repeated measures over the years.

**Results:**

The median age was 66 years, median BMI was 24.56 kg/m^2^, and median eGFR was 89.86 mL/min.1.73 m^2^. For every 1-year increase in age, women’s eGFR decreased by 1.06 mL/min/1.73 m^2^ and men’s by 0.91 mL/min/1.73 m^2^. We observed greater age-related eGFR decline in men and women with high systolic blood pressure (SBP). Men with high triglyceride (TG), high low-density lipoprotein cholesterol (LDL-C), and low high-density lipoprotein cholesterol (HDL-C), had greater age-related eGFR decline. In women, different BMI groups showed significant differences in age-related eGFR decline, with the highest decline in those with obesity. Additionally, participants with normal baseline eGFR had a faster age-related decline than those with low baseline eGFR.

**Conclusion:**

The eGFR declined linearly with age in Chinese older adults, with women exhibiting a slightly faster decline than men. Both men and women should be cautious of SBP. Older adults with normal baseline renal function experienced a faster eGFR decline. Men with high TG, LDL-C, and low HDL-C levels, as well as obese women, should be vigilant in monitoring renal function.

## 1 Introduction

The global population’s aging is the most important medical and social demographic problem worldwide (1). In recent decades, increased average life expectancy has led to a higher proportion of older adults individuals worldwide, accompanied by a rise in non-communicable diseases, including chronic kidney disease (CKD). Aging is a progressive and inevitable biological process, characterized by structural and functional changes in all organs, including the kidneys. The decline in kidney function with advancing age primarily manifests as a decrease in glomerular filtration rate (GFR) (2, 3), the most widely used parameter for measuring kidney function. Although the decrease in GFR with aging has been recognized, the exact estimation of the magnitude of the renal function decline with healthy aging is not yet well-established.

Most studies (4, 5) investigating GFR decline in older adults have relied on cross-sectional designs, which may complicate interpretation due to interindividual modeling across different age groups, disregarding individual trajectories over time.

As the previous study confirmed (6), the generation change in the cross-sectional study and the eGFR decline rate in the longitudinal study are different. Moreover, few studies have included community-dwelling individuals, limiting their ability to reflect real-world decline in a representative older adult population (7). Longitudinal studies with repeated eGFR measurements to model age-related trajectory in old age are scarce. Our research tried to fill the data gap of the course of renal function decline in the older adults population in China, which country has the world’s largest older population (8). Gold standard GFR measurement methods, such as inulin clearance or plasma clearance using radiolabeled or non-radiolabeled exogenous markers, are laborious, expensive, and not feasible for routine clinical use (9). Therefore, estimated glomerular filtration rate (eGFR) is a more accessible approach for evaluating kidney function in daily clinical practice.

Considering the frequent comorbidities, such as obesity (10), diabetes (11, 12) and cardiovascular diseases (13), prevalent in the geriatric population and their potential impact on kidney function, we conducted further analyses to examine changes in renal function among the older adults across different health states with increasing age. Investigating the interaction between health status and age on the renal function decline among the older adults is essential for developing targeted interventions that enhance health status and quality of life, promoting healthy aging.

## 2 Materials and methods

### 2.1 Study cohort

**Figure 1:**
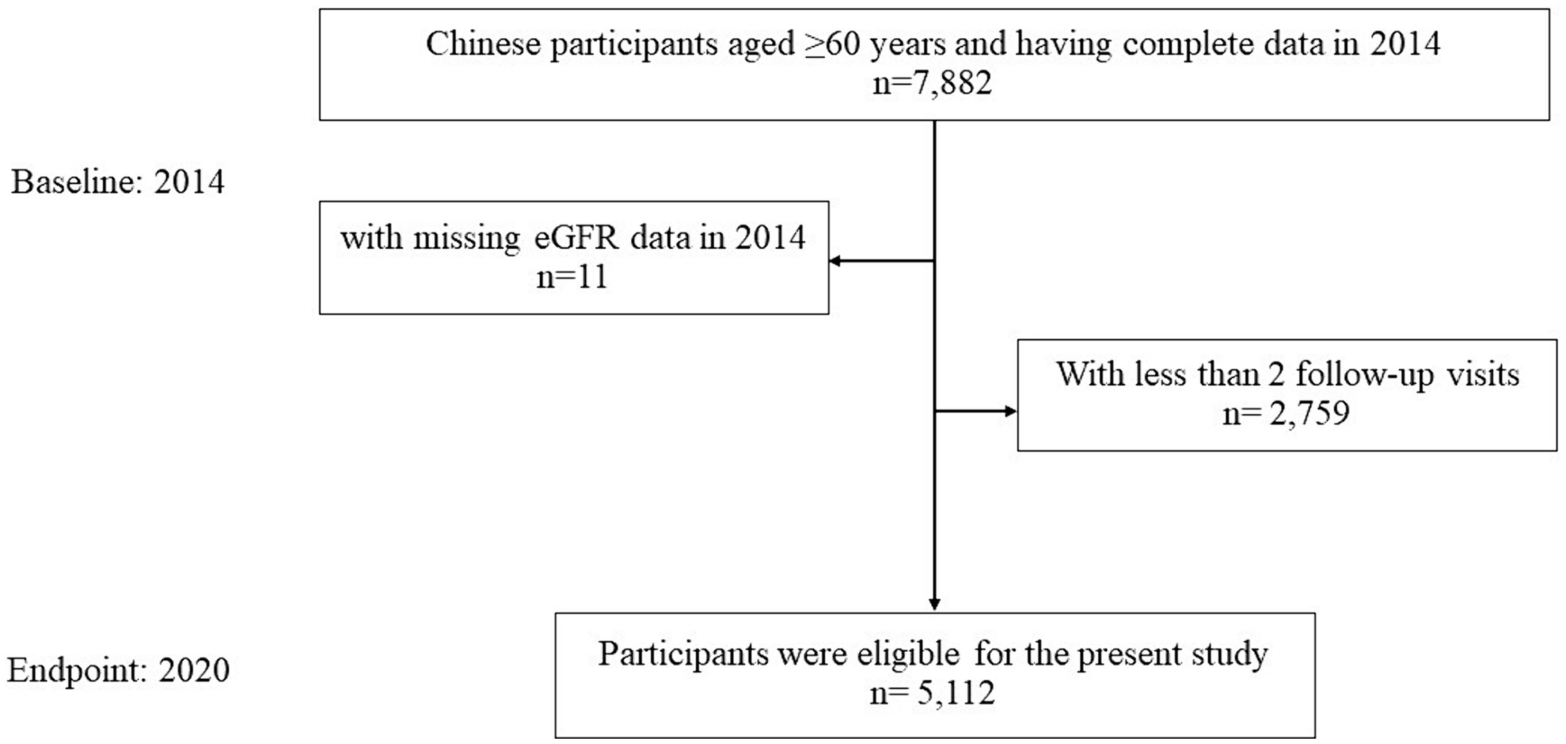
Flow chart for participants selection.

This study was an observational, retrospective, single-center cohort study, which derived from a total number of 7,871 Chinese older participants who had received a health check at the Health Management Center of Ren Ji hospital in year 2014, with available eGFR information. Participants were followed annually to 2020. After excluding participants with less than 1 follow-up visits (n = 2,759) after 2014, the sample size was 5,112 Chinese older adults (3,321 men and 1,791 women, range 60–104 years), with 22,072 observations (Figure 1). Throughout the study period, the proportion of missing participants in annual follow-up examinations increased progressively, starting from baseline as follows: 0, 18.6, 30.6, 42.2, 51.2, 59.5, 66.1%. Notably, 1,059 participants completed every follow-up throughout the entire cohort study period.

### 2.2 Estimated glomerular filtration rate

All biochemical tests were performed on venous blood taken after fasting for at least 8 h. The eGFR was calculated by the CKD-EPI equation based on age, sex, and serum creatinine concentration (14). We annually measured serum level of creatinine for each participant through the study (2014–2020).

### 2.3 Clinical information

Clinical information was abstracted from medical records. In brief, height and body weight were measured by trained nurses. According to body mass index (BMI), participants were classified as underweight (< 18.5 kg/m^2^), normal weight (18.5 to 23.9 kg/m^2^), overweight (24.0 to 27.9 kg/m^2^), or obese (≥ 28.0 kg/m^2^) based on Chinese criteria (15). Blood pressure was measured using an automatic blood pressure monitor (HBP-9020, Omron, China) after at least 10-min rest. Venous blood samples were drawn in the morning after participants were fasted for at least 8 h. All the blood samples were analyzed in the same clinical laboratory center. Serum creatinine was analyzed using the enzymatic method by Roche (CREP2; Roche Diagnostics, Mannheim, Germany), fasting blood glucose (FBG) was analyzed using the hexokinase method, total cholesterol (TC) and total triglycerides (TG) were analyzed using the enzymatic colorimetric method, high-density lipoprotein cholesterol (HDL-C) and low-density lipoprotein cholesterol (LDL-C) were analyzed using the homogeneous enzymatic colorimetric assay. These six indicators were analyzed on Roche Cobas C701 module. Hemoglobin was measured by an automatic hematology analyzer (XN-10, Sysmex, Japan). The classified criteria were as follows: systolic blood pressure (SBP) ≥ 140 mmHg (high SBP), diastolic blood pressure (DBP) ≥ 90 mmHg (high DBP) (16), FBG ≥ 7.0 mmoL/L (high FBG) (17), TC ≥ 6.2 mmol/L (high TC), TG ≥ 2.3 mmol/L (high TG), LDL-C ≥ 4.1 mmol/L (high LDL-C), HDL-C < 1.0 mmol/L (low HDL-C) (18), eGFR <60 mL/min/1.73 m^2^ (low eGFR). Anemia was diagnosed if hemoglobin was <130 g/L in men while <120 g/L in women (19).

### 2.4 Statistical analysis

In the descriptive statistics, continuous variables were tested for normal distribution, and if deviating, represented using medians with interquartile ranges (25th-75th quartile). Categorical variables were shown as percentage. The individual rate of eGFR decline (i.e., the slope; ml/min/1.73m^2^/year) was analyzed using linear mixed-effects model to account for repeated measures over the years. Missing data were accounted for by using the mixed-effects model (20).

To quantify the age-dependent eGFR, our main analyses tested the association between age and eGFR, while omitting the interaction between age and follow-up time. This was because there was no interaction between age and follow-up duration observed. In contrast, as noticing a significant difference between the sexes, all the analyses were performed among men and women separately (Table 1). We then tested the additive interaction between age and the baseline health status, indicated by other biomarkers on eGFR (Table 2). In the end, sensitivity analyses were conducted to repeat the abovementioned analyses on the 1,059 participants who had undergone all follow-ups, for testing the existence of selection bias.

**Table 1:** Age-related change of eGFR (ml/min/1.73 m^2^) in 5,112 Chinese older adults during the whole cohort study.

Sex	2014 (baseline)	2015	2016	2017	2018	2019	2020	Slope _age_
Men (*n* = 3,321)	Ref	−1.06 (−1.33, −0.80)	−4.87 (−5.16, −4.57)	−2.21 (−2.55, −1.86)	−3.00 (−3.42, −2.59)	−3.08 (−3.54, −2.62)	−3.80 (−4.34, −3.26)	−0.91 (−0.95, −0.86)
Women (*n* = 1,791)	Ref	−2.01 (−2.37, −1.65)	−6.52 (−6.96, −6.07)	−3.20 (−3.64, −2.76)	−4.34 (−4.85, −3.83)	−4.19 (−4.80, −3.59)	−5.07 (−5.78, −4.37)	−1.06 (−1.12, −0.99)

The data was analyzed by linear mixed-effects model with random intercept. The data under each natural year displayed the change value of mean eGFR (95% confidence interval) in that year compared with the baseline. The last column Slope _age_ represented the slope of eGFR decline during the whole study period fitted by the linear mixed-effects model, with age measured in years.

**Table 2:** Additive interactions between baseline health indicators and age on eGFR among 5,112 Chinese older adults.

Stratified variables	Men (*n* = 3,321)			Women (*n* = 1,791)		
	*N*	Beta-estimate for slope	*p* for interaction	*N*	Beta-estimate for slope	*p* for interaction
High SBP	1,386	−0.95 (−1.01, −0.89)	0.01	761	−1.13 (−1.22, −1.05)	0.02
Normal SBP	1834	−0.85 (−0.91, −0.79)		923	−1.00 (−1.09, −0.92)	
High DBP	603	−0.97 (−1.06, −0.87)	0.16	185	−1.08 (−1.26, −0.91)	0.82
Normal DBP	2,617	−0.90 (−0.95, −0.85)		1,499	−1.06 (−1.13, −0.99)	
Underweight	74	−0.67 (−0.91, −0.42)	0.24	34	−1.00 (−1.36, −0.63)	0.01
Normal weight	1,103	−0.90 (−0.97, −0.84)		732	−0.96 (−1.05, −0.87)	
Overweight	1,401	−0.92 (−0.98, −0.85)		607	−1.08 (−1.18, −0.97)	
Obesity	384	−0.93 (−1.05, −0.81)		220	−1.26 (−1.41, −1.10)	
High FBG	368	−0.91 (−1.03, −0.80)	0.88	170	−1.14 (−1.30, −0.97)	0.34
Normal FBG	2,952	−0.90 (−0.95, −0.86)		1,621	−1.06 (−1.12, −0.99)	
High TC	227	−1.05 (−1.21, −0.90)	0.06	342	−1.08 (−1.20, −0.95)	0.64
Normal TC	2,796	−0.90 (−0.95, −0.85)		1,390	−1.05 (−1.12, −0.97)	
High TG	493	−1.08 (−1.18, −0.97)	< 0.001	297	−1.11 (−1.24, −0.98)	0.32
Normal TG	2,828	−0.89 (−0.94, −0.84)		1,494	−1.04 (−1.11, −0.98)	
High LDL-C	197	−1.20 (−1.37, −1.04)	< 0.001	250	−1.03 (−1.18, −0.89)	0.73
Normal LDL-C	3,098	−0.89 (−0.94, −0.84)		1,526	−1.06 (−1.13, −0.99)	
High HDL-C	540	−1.03 (−1.13, −0.93)	0.008	106	−1.08 (−1.28, −0.88)	0.78
Normal HDL-C	2,755	−0.89 (−0.94, −0.84)		1,671	−1.05 (−1.12, −0.98)	
Anemia	209	−0.84 (−0.98, −0.70)	0.77	106	−1.17 (−1.35, −0.99)	0.11
Non-anemia	3,110	−0.86 (−0.91, −0.81)		1,684	−1.02 (−1.08, −0.95)	
Low eGFR	191	−0.14 (−0.27, −0.005)	< 0.001	59	0.04 (−0.20, 0.28)	< 0.001
Normal eGFR	3,130	−0.70 (−0.74, −0.66)		1732	−0.85 (−0.91, −0.79)	

High SBP: systolic blood pressure ≥ 140 mmHg. High DBP: diastolic blood pressure ≥ 90 mmHg. High TC: total cholesterol ≥ 6.2 mmol/L. High TG: triglyceride ≥ 2.3 mmol/L. High LDL-C: low-density lipoprotein cholesterol ≥ 4.1 mmol/L. Low HDL-C: high-density lipoprotein cholesterol < 1.0 mmol/L. Low eGFR: estimated glomerular filtration rate < 60 mL/min/1.73 m^2^. Participants were classified into four BMI groups: underweight (BMI < 18.5 kg/m^2^), normal weight (18.5 kg/m^2^ ≤ BMI < 24 kg/m^2^), overweight (24 kg/m^2^ ≤ BMI < 28 kg/m^2^), and obesity (BMI ≥ 28 kg/m^2^). High FBG: fasting blood glucose ≥ 7.0 mmol/L.Anemia in men: hemoglobin < 130 g/L; women: hemoglobin < 120 g/L. The data were analyzed by linear mixed-effects model with random intercept.

All statistical analyses were performed with SAS version 9.4 (SAS Institute Inc., Cary, North Carolina). Confidence intervals (CI) were determined at 95% level.

## 3 Results

### 3.1 Main baseline characteristics

Among 5,112 participants in 2014, mean eGFR was 87.88 (77.36, 94.43) ml/min/1.73 m2 among men and 92.99 (84.03, 97.46) ml/min/1.73 m^2^ among women. The baseline characteristics were presented in Table 3. Baseline characteristics of the study participants showed that men had higher serum creatinine levels, lower eGFR, lower levels of total cholesterol, triglycerides, LDL-C, and HDL-C compared to women. Slightly higher DBP and FBG were observed in males, but no significant difference was found in SBP. The prevalence of underweight and obesity was similar between males and females, but the rate of overweight was higher in males (47.3% versus 38.1%) compared to females.

**Table 3:** Baseline characteristics of 5,112 Chinese older adults.

Characteristics	Total (*n* = 5,112)	Men (*n* = 3,321)	Women (*n* = 1,791)	*p* value
Age (years)	66 (62, 73)	66 (62, 74)	65 (62, 72)	< 0.001
BMI (kg/m^2^)	24.56 (22.48, 26.53)	24.69 (22.68, 26.57)	24.14 (22.13, 26.45)	< 0.001
SBP (mmHg)	137 (124, 149)	136 (124, 148)	137 (124, 150)	0.40
DBP (mmHg)	79 (71, 86)	77.2 (69.2, 86.8)	76 (68, 83)	< 0.001
Creatinine (μmol/L)	70.6 (60.0, 81.9)	76.2 (68.6, 84.9)	57.2 (51.1, 64.0)	< 0.001
eGFR (ml/min/1.73 m^2^)	89.86 (79.73, 95.68)	87.88 (77.36, 94.43)	92.99 (84.03, 97.46)	< 0.001
TC (mmol/L)	5.06 (4.44, 5.71)	4.87 (4.26, 5.48)	5.39 (4.81, 6.05)	< 0.001
TG (mmol/L)	1.33 (0.97, 1.90)	1.31 (0.95, 1.88)	1.35 (1.01, 1.94)	0.003
LDL-C (mmol/L)	2.99 (2.46, 3.51)	2.88 (2.36, 3.41)	3.17 (2.67, 3.74)	< 0.001
HDL-C (mmol/L)	1.32 (1.12, 1.58)	1.25 (1.06, 1.49)	1.47 (1.25, 1.72)	< 0.001
FBG (mmol/L)	5.33 (4.96, 5.90)	5.40 (5.00, 5.99)	5.30 (4.90, 5.84)	< 0.001
Hemoglobin (g/L)	144 (134, 154)	150 (142, 158)	134 (128, 140)	< 0.001
Underweight (*n*)	2.37% (108)	2.50% (74)	2.13% (34)	0.44
Overweight (*n*)	44.08% (2,008)	47.30% (1,401)	38.10% (607)	< 0.001
Obesity (*n*)	13.26% (604)	12.96% (384)	13.81% (220)	0.42

BMI, body mass index; SBP, systolic blood pressure; DBP, diastolic blood pressure; eGFR, estimated glomerular filtration rate; FBG, fasting blood glucose; TC, total cholesterol; TG, triglyceride; LDL-C, low-density lipoprotein cholesterol; HDL-C, high-density lipoprotein cholesterol. The difference between men and women was tested by Wilcoxon text. Variables were presented as medians with interquartile ranges (25th–75th percentile).

### 3.2 Age-related change of eGFR

**Figure 2:**
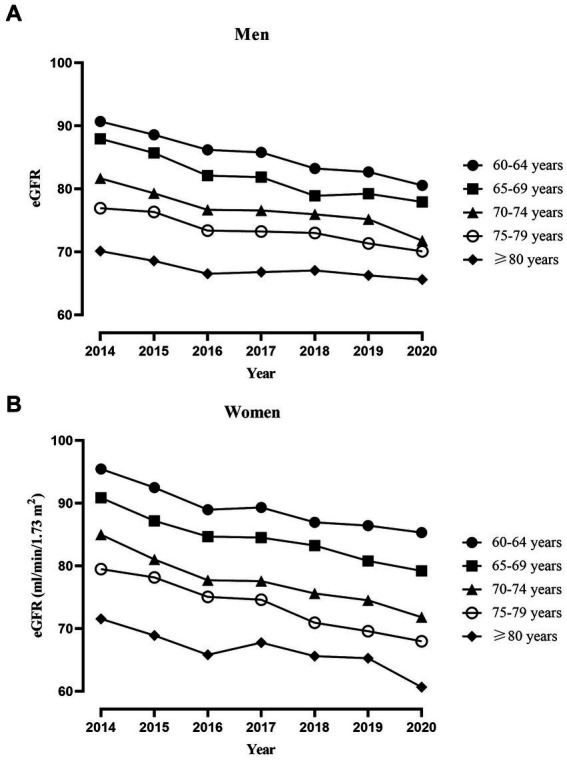
Trajectory of average eGFR in different age groups of older adults individuals throughout the study period. **(A)** Trajectory of average eGFR in 3,321 men. **(B)** Trajectory of average eGFR in 1,791 women.

The slope of age obtained from the linear mixed-effects model represents the change in eGFR for every 1-year increase in age. As shown in Table 1, for every 1-year increase in age, eGFR decreased by 1.06 mL/min/1.73 m^2^ (0.99, 1.12) in women, while it decreased by 0.91 mL/min/1.73 m^2^ (0.86, 0.95) in men. Specific trajectory according to age groups were presented in Figure 2. We analyzed eGFR decline in healthy older adults without hypertension, hyperglycemia, dyslipidemia, low eGFR, and obesity. In line with the main analysis, the declining trend in the healthy older adults appears to be consistent, albeit at a slower rate of decline (Supplementary Table 1).

### 3.3 Additive interactions between health indicators and age on eGFR

In Table 2, we observed greater age-related eGFR decline in men and women with high SBP compared to normal SBP (*p interaction = 0.01* and *0.02*). Men with high TG and high LDL-C also had greater age-related eGFR decline compared to normal TG and LDL-C (both *p interaction < 0.001*). Additionally, men with low HDL-C had larger age-related eGFR decline compared to normal HDL-C (*p interaction = 0.006*). In women, there was a significant difference in the age-related decline of eGFR among different BMI groups, with the highest decline observed in obese women and the slowest decline observed in women with normal weight (*p interaction = 0.01*). Furthermore, there was no significant difference in age-related decline of eGFR between anemic individuals, irrespective of gender, and non-anemic individuals. In both men and women with normal baseline eGFR, the age-related decline was significantly faster than in those with low baseline eGFR.

### 3.4 Sensitivity analyses

The sensitivity analysis of 1,059 participants who completed every follow-up revealed that estimated values in Supplementary Table 2 closely mirrored those in the main analyses. Confidence intervals in Supplementary Table 3 consistently overlapped with the main analyses. Discrepancies in *p*-values across certain groups may be attributed to varying sample sizes, with selection bias less likely to influence outcomes.

## 4 Discussion

We investigated the course of kidney function decline in the cohort of older participants using repeat GFR estimates and observed the following main findings. First, we found that the eGFR, determined by serum creatinine, declined linearly with age in Chinese older adults. For every 1-year increase in age, eGFR decreased by 1.06 mL/min/1.73 m^2^ (0.99, 1.12) in women, while it decreased by 0.91 mL/min/1.73 m^2^ (0.86, 0.95) in men.

The discovery of linear decline was like the longitudinal German population-based cohort in persons aged 70 and above with a repeated estimation of GFR over a median of 6.1 years of follow-up (7). The possible reason is that aging leads to notable kidney structural and functional changes, even without age-related health issues. Kidney cortical volume decreases, surface roughness increases, and simple renal cysts grow with age. Additionally, histologic signs of nephrosclerosis increase with age, as does the decline in nephron number and whole-kidney GFR (21). Moreover, a recent longitudinal analysis of older adults stage 4 and 5 CKD showed similar linear decline, but opposite gender differences compared to our study (22). This difference may be due to higher comorbidity burden and sex differences in advanced CKD patients. For instance, male participants had twice the myocardial infarction rate of female participants. One of the possible reasons for the gender difference may be caused by a sex-dependent decrease in muscle mass with age, biasing our eGFRs.

Second, we found that older adults individuals with poor baseline renal function experienced a slower decline in age-related glomerular filtration rate compared to those with normal renal function. A large Japanese longitudinal study revealed for the first time that eGFR decline rate in healthy subjects depended mainly on eGFR at baseline, but not on age (6). The results of this study supported our findings, which were different from those of previous studies. For example, a study more than 10 years ago showed that eGFR decline was faster with a lower baseline eGFR (23). The discrepancy in research findings was primarily attributed to the previous study measuring serum creatinine only twice over a ten-year period, while our study conducted annual measurements for 6 years. Furthermore, distinct fitting models were utilized for statistical analysis. The reason why the rate of eGFR decline was slower with a lower baseline eGFR is unclear, but a compensatory mechanism might work as kidney function decreases.

Furthermore, we observed greater age-related eGFR decline both in men and women with high SBP compared to normal SBP. A Japanese cohort study also showed that a difference in SBP, but not DBP, is independently associated with a rapid eGFR decline in the general Japanese population, including older subjects (24). Various mechanisms might contribute to kidney injuries in older adults hypertensive patients, including the renin-angiotensin-aldosterone system, oxidative stress, endothelial dysfunction, and genetic and epigenetic factors (25).

Finally, we observed that men with high triglyceride, high LDL-C, or low HDL-C had a faster age-related eGFR decline. Additionally, in BMI groups, obese women experienced the fastest age-related eGFR decline. Other studies (26, 27) also found that high triglyceride and low HDL-C predicted renal function decline. A 7-year cohort study of the older adults found that baseline BMI was associated with an increased risk of rapid eGFR loss (28). Pathways through which obesity might cause renal damage were not well understood. Potential mechanisms included sympathetic nervous and renin-angiotensin-aldosterone systems activation, mechanical stress, hormonal imbalance, and production of inflammatory cytokines (29). Some scholars proposed that renal lipid accumulation could cause structural and functional changes in mesangial cells, podocytes, and proximal tubule cells, affecting nephron function (30). Irrespective of the underlying pathophysiological mechanisms, it was crucial for older adults with obesity or hyperlipidemia to closely monitor their renal function. Further research and clinical trials were needed to assess the efficacy of these interventions in this vulnerable population.

The study’s longitudinal design with a large cohort and multiple biomarkers enhances its uniqueness. It improves our understanding of age-related kidney function decline and provides valuable insights into eGFR decline patterns in old age. It addresses the data gap on renal function decline in China’s older adults population across different health conditions. This study has practical implications for clinical practice and future gerontology research, facilitating the development of targeted interventions to enhance the health status and quality of life of the older adults, promoting healthy aging.

Our study did not include the endogenous biomarker cystatin C, which is potentially more suitable for assessing renal function in older individuals compared to creatinine, as creatinine levels can be affected by sarcopenia (31). This retrospective observational study may include both healthy and sick individuals. Limited data on proteinuria (24), etiological diagnosis of CKD, comorbidities (10, 12, 13), and medication (32) makes it impossible to exclude the impact of diseases and medication on renal function. These uncontrolled confounding factors restrict the generalizability of the study results. Additionally, there may be other factors, such as socioeconomic status (33), dietary habits (34, 35), and physical activity (36), that could influence the risk of renal function decline. However, information regarding these factors was not available in our study.

## 5 Conclusion

The eGFR, determined by serum creatinine, declined linearly with age in Chinese older adults. Women exhibited a slightly faster age-related decline in eGFR compared to men. Additionally, older adults with good baseline renal function experienced a more rapid eGFR decline than those with lower baseline eGFR. Both men and women should be cautious of SBP. Men with high TG, LDL-C, and low HDL-C levels, as well as obese women, should be vigilant in monitoring renal function.

## Data availability statement

The raw data supporting the conclusions of this article will be made available by the authors, without undue reservation.

## Ethics statement

The studies involving humans were approved by Ethical Committee of Ren Ji Hospital. The studies were conducted in accordance with the local legislation and institutional requirements. The participants provided their written informed consent to participate in this study.

## Author contributions

YJ: Writing – review & editing, Writing – original draft, Visualization, Software, Methodology, Formal analysis, Data curation, Conceptualization. QC: Writing – review & editing, Validation, Supervision, Resources, Project administration, Investigation, Data curation. WH: Writing – review & editing, Writing – original draft, Visualization, Software, Methodology, Formal analysis, Conceptualization. TX: Writing – review & editing, Writing – original draft, Software, Methodology, Data curation. MT: Writing – original draft, Visualization, Software, Methodology, Data curation. YL: Writing – review & editing, Visualization, Supervision, Conceptualization. RX: Writing – review & editing, Visualization, Validation, Supervision, Software, Resources, Project administration, Methodology, Funding acquisition, Conceptualization.
