# Supplementary material for: Age and estimated glomerular filtration rate in Chinese older adults: a cohort study from 2014 to 2020
Source: Front Public Health. 2024 Jun 25;12:1392903. doi: 10.3389/fpubh.2024.1392903 (PMC11231386; doi:10.3389/fpubh.2024.1392903)
Supplement: Supplementary file 1 [file Table_1.docx]

**Supplementary Table 1. The slope (ml/min/1.73 m^2^/year) of eGFR decline during the whole cohort study.**

|  | Men | | Women | |
| --- | --- | --- | --- | --- |
| Baseline disease | N | slope | N | slope |
| Healthy | 1,058 | -0.65 (-0.72, -0.59) | 616 | -0.85 (-0.95, -0.75) |
| Hypertension | 1,503 | -0.94 (-1.01, -0.88) | 786 | -1.12 (-1.22, -1.02) |
| Without hypertension | 1,718 | -0.85 (-0.91, -0.79) | 898 | -1.02 (-1.12, -0.93) |
| Hyperglycemia | 368 | -0.84 (-1.00, -0.67) | 170 | -0.92 (-1.11, -0.74) |
| Without hyperglycemia | 2,952 | -0.91 (-0.96, -0.86) | 1,621 | -1.07 (-1.14, -1.01) |
| Obesity | 384 | -0.98 (-1.14, -0.82) | 220 | -1.23 (-1.44, -1.02) |
| Without obesity | 2,578 | -0.88 (-0.93, -0.83) | 1,373 | -1.02 (-1.10, -0.94) |
| Dyslipidemia | 1,034 | -1.03 (-1.12, -0.94) | 620 | -1.02 (-1.13, -0.91) |
| Without dyslipidemia | 2,287 | -0.88 (-0.93, -0.82) | 1,171 | -1.07 (-1.15, -0.99) |

Note:

1. The data was analyzed by linear mixed-effects model with random intercept.

2. Hypertension was defined as systolic blood pressure (SBP) ≥140 mmHg and/or diastolic blood pressure (DBP) ≥90 mmHg. Hyperglycemia was defined as FPG ≥7.0 mmol/L. Obesity was defined as BMI ≥ 28 kg/m^2^. Dyslipidemia was defined as total cholesterol ≥ 6.2 mmol/L or triglyceride ≥ 2.3 mmol/L or low-density lipoprotein cholesterol ≥ 4.1 mmol/L or high-density lipoprotein cholesterol < 1.0 mmol/L.

3. Healthy participants are defined as those who do not exhibit hypertension, hyperglycemia, obesity, or chronic kidney disease (eGFR < 60 ml/min/1.73 m^2^).

**Supplementary Table 2. Sensitivity analyses of age-related changes in eGFR (ml/min/1.73 m^2^) among 1,059 participants who completed all follow-ups.**

| Sex | 2014 (baseline) | 2015 | 2016 | 2017 | 2018 | 2019 | 2020 | Slope _age_ |
| --- | --- | --- | --- | --- | --- | --- | --- | --- |
| Men  (n= 670) | ***Ref*** | -0.63  (-1.16, -0.09) | -4.73  (-5.29, -4.16) | -2.51  (-3.12, -1.90) | -2.83  (-3.55, -2.12) | -2.73  (-3.52, -1.94) | -3.54  (-4.44, -2.65) | -0.87  (-0.97, -0.77) |
| Women  (n=389) | ***Ref*** | -1.45  (-2.14, -0.76) | -6.33  (-7.11, -5.55) | -3.00  (-3.85, -2.14) | -4.04  (-4.98, -3.10) | -3.83  (-4.91, -2.74) | -4.82  (-6.08, -3.57) | -1.09  (-1.23, -0.95) |

Note:

1. The data was analyzed by linear mixed-effects model with random intercept.

2. The data under each natural year displayed the change value of mean eGFR (95% confidence interval) in that year compared with the baseline.

3. The last column Slope _age_ represented the slope of eGFR decline during the whole study period fitted by the linear mixed-effects model, with age measured in years.

**Supplementary Table 3. Sensitivity analyses of additive interactions between baseline health indicators and age on changes of eGFR (ml/min/1.73 m^2^) among 1,059 participants who completed all follow-ups.**

| Stratified variables | Men (n= 670) | | | Women (n= 389) | | |
| --- | --- | --- | --- | --- | --- | --- |
|  | N | Beta-Estimate for slope | p for interaction | N | Beta-Estimate for slope | p for interaction |
| High SBP | 292 | -0.89 (-1.02, -0.77) | 0.42 | 151 | -1.11 (-1.29, -0.94) | 0.47 |
| Normal SBP | 359 | -0.84 (-0.96, -0.71) |  | 220 | -1.04 (-1.22, -0.86) |  |
| High DBP | 131 | -0.94 (-1.12, -0.77) | 0.26 | 36 | -1.02 (-1.34, -0.69) | 0.63 |
| Normal DBP | 520 | -0.85 (-0.95, -0.74) |  | 335 | -1.10 (-1.25, -0.95) |  |
| Underweight | 11 | -0.46 (-1.00, 0.08) | 0.23 | 7 | -1.58 (-2.28, -0.87) | 0.17 |
| Normal weight | 212 | -0.79 (-0.93, -0.64) |  | 162 | -1.06 (-1.24, -0.87) |  |
| Overweight | 278 | -0.89 (-1.02, -0.75) |  | 140 | -1.09 (-1.29, -0.90) |  |
| Obesity | 69 | -0.94 (-1.18, -0.70) |  | 36 | -1.39 (-1.75, -1.03) |  |
| High FBG | 69 | -1.01 (-1.24, -0.77) | 0.17 | 30 | -1.23 (-1.59, -0.87) | 0.40 |
| Normal FBG | 600 | -0.85 (-0.95, -0.75) |  | 359 | -1.07 (-1.22, -0.93) |  |
| High TC | 36 | -1.13 (-1.45, -0.81) | 0.06 | 75 | -1.10 (-1.34, -0.86) | 0.94 |
| Normal TC | 530 | -0.83 (-0.95, -0.71) |  | 293 | -1.11 (-1.27, -0.95) |  |
| High TG | 109 | -0.99 (-1.18, -0.80) | 0.13 | 67 | -1.24 (-1.49, -0.99) | 0.17 |
| Normal TG | 560 | -0.84 (-0.95, -0.74) |  | 320 | -1.06 (-1.21, -0.91) |  |
| High LDL-C | 33 | -1.12 (-1.45, -0.79) | 0.11 | 59 | -1.08 (-1.36, -0.79) | 0.92 |
| Normal LDL-C | 633 | -0.86 (-0.96, -0.75) |  | 328 | -1.09 (-1.24, -0.94) |  |
| High HDL-C | 106 | -1.07 (-1.25, -0.89) | 0.02 | 18 | -1.66 (-2.16, -1.15) | 0.02 |
| Normal HDL-C | 528 | -0.85 (-0.96, -0.74) |  | 364 | -1.07 (-1.21, -0.92) |  |
| Anemia | 32 | -0.81 (-1.12, -0.50) | 0.78 | 18 | -1.64 (-2.11, -1.17) | 0.02 |
| Non-anemia | 638 | -0.85 (-0.96, -0.75) |  | 371 | -1.07 (-1.22, -0.93) |  |
| Low eGFR | 25 | -0.49 (-0.80, -0.18) | 0.14 | 9 | -0.68 (-1.33, -0.03) | 0.43 |
| Normal eGFR | 645 | -0.73 (-0.82, -0.64) |  | 380 | -0.94 (-1.07, -0.80) |  |

Note:

1. High SBP: systolic blood pressure ≥ 140 mmHg. High DBP: diastolic blood pressure ≥ 90 mmHg.

2. High TC: total cholesterol ≥ 6.2 mmol/L. High TG: triglyceride ≥ 2.3 mmol/L. High LDL-C: low-density lipoprotein cholesterol ≥ 4.1 mmol/L. Low HDL-C: low-density lipoprotein cholesterol < 1.0 mmol/L.

3. Low eGFR: estimated glomerular filtration rate < 60 ml/min/1.73 m^2^.

4. Participants were classified into four BMI groups: underweight (BMI < 18.5 kg/m^2^), normal weight (18.5 kg/m^2^ ≤ BMI < 24 kg/m^2^), overweight (24 kg/m^2^ ≤ BMI < 28 kg/m^2^), and obesity (BMI ≥ 30 kg/m^2^).

5. High FBG: fasting blood glucose≥ 7.0 mmol/L.

6. Anemia in men: hemoglobin < 130 g/L, women: hemoglobin < 120 g/L.

7. The data were analyzed by linear mixed-effects model with random intercept.
